# Efficacy and feasibility of ambulatory treatment-based monthly nedaplatin plus S-1 in definitive or salvage concurrent chemoradiotherapy for early, advanced, and relapsed esophageal cancer

**DOI:** 10.1186/s13014-016-0587-9

**Published:** 2016-01-19

**Authors:** Hideomi Yamashita, Akihiro Haga, Ryousuke Takenaka, Tomoki Kiritoshi, Kae Okuma, Kuni Ohtomo, Keiichi Nakagawa

**Affiliations:** Department of Radiology, University of Tokyo Hospital of Bunkyo-ku, Tokyo, Japan

**Keywords:** Esophageal cancer, Definitive chemoradiotherapy, Nedaplatin, S-1

## Abstract

**Background:**

Standard chemoradiotherapy (CRT) using cisplatin (CDDP) and 5-fluorouracil (5-FU) is an optional treatment for patients with stage II-III esophageal cancer. However, there are some demerits in this regimen because CDDP administration requires a large transfusion volume and 5-FU must be continuously infused over 24 h. Therefore, hospitalization is unavoidable. We collected retrospectively the data of definitive CRT with nedaplatin and S-1 as carried out in our institution.

**Methods:**

Patients with early and advanced esophageal cancer and relapsed esophageal cancer after radical surgery were included. Nedaplatin 80 mg/m^2^ was given on days 1 and 29, and S-1 80 mg/m^2^ on days 1-14 and 29-42. No prophylactic treatment with granulocyte colony stimulating factor was administered. Patients received two courses of concurrent radiotherapy of more than 50 Gy with or without two additional courses as adjuvant therapy every 4 weeks.

**Results:**

Between August 2011 and June 2015, 89 patients (age range, 44–86 years; K-PS 90–100, 81 %; squamous cell carcinoma histology, 97 %; definitive/salvage CRT, 75/25 %) were collected. Twenty-one (24 %) patients completed four cycles, and 94 % received two or more cycles. Grade 4 leukopenia, thrombocytopenia, and anemia occurred in 12, 7, and 10 % of the patients, respectively. Five patients developed febrile neutropenia. Grade 3 non-hematological toxicity included infection in 12 %, mucositis/esophagitis in 3 %, kidney in 3 %, and fatigue in 3 %. Sixty-four patients (72 %) received the prescribed full dose and full cycles of chemotherapy. A complete response was achieved in 76 patients (85 %). The 3-year overall survival rate was 54.4 % in definitive CRT and 39.8 % in salvage CRT, respectively. Sixty-two subjects (70 %) received treatment as outpatients.

**Conclusions:**

Nedaplatin and S-1 in combination with radiotherapy is feasible, and toxicity is tolerable. This treatment method has the potential to shorten hospitalization without impairing the efficacy of CRT.

## Introduction

Concurrent chemoradiotherapy (CRT) is well established as a standard approach to treat esophageal cancer.

Cis-diammine-glycolatoplatinum (nedaplatin) is a platinum derivative that was developed with the aim of reducing renal toxicity while maintaining the effectiveness of CDDP [1]. In an in vivo study, a combination of nedaplatin (NDP) and 5-FU has been shown to be as effective as a combination of CDDP and 5-FU [2]. In a clinical study, combination chemotherapy using NDP and 5-FU has been reported to be a safe and effective method for treating advanced esophageal cancer [3, 4].

S-1 (TS1 ®; Taiho Pharmaceutical Co. Ltd., Tokyo, Japan), a new biochemical modulator of 5-FU, is an oral dihydropyrimidine dehydrogenase (DPD) inhibitory fluoropyrimidine based on the biochemical modulation of 5-FU [5–7]. The advantages of S-1 compared with 5-FU are greater convenience because of its oral formulation and continuous delivery, without intravenous infusion. S-1 is frequently used as a substitute for 5-FU in gastric cancer, but limited data are available for esophageal cancer [8]. Recently, combination chemotherapy with S-1 and cisplatin has been widely studied in advanced gastric cancer [9–11].

To our knowledge, there are no reports published on definitive CRT with S-1 and NDP in patients with resectable esophageal cancer. We designed this study as a pilot study using concurrent CRT with NDP and S-1 in early, advanced, and recurrent esophageal cancer. From the tolerability and clinical efficacy of this regimen, we evaluated retrospectively the possibility of introducing this new chemotherapeutic regimen, with concurrent radiation therapy, in the treatment of esophageal cancer.

## Patients and methods

### Chemotherapy regimen

All patients received chemotherapy concurrent with irradiation. Chemotherapy consisted of two cycles of S-1 (80 mg/m^2^/day, days 1–14 and days 29–42, continuously) combined with NDP (80 mg/m^2^, day 1 and day 29, bolus); standard techniques were used for hydration and alkalization. For patients 75 years old or older, doses were reduced to 80 % of both S-1 and NDP without adjuvant chemotherapy. Chemotherapy was started on the first day of irradiation. After concurrent CRT, in the adjuvant setting one or two cycles of the same dose of chemotherapy were added for patients who still had sufficient bone-marrow function and performance status and who did not refuse additional chemotherapy, although old patients and patients with stage I disease were excluded. When grade 4 myelosuppression was seen and patients had recovered, doses were reduced to 80 % dose of both NDP and S-1 in the subsequent cycle or later.

**Fig. 1:**
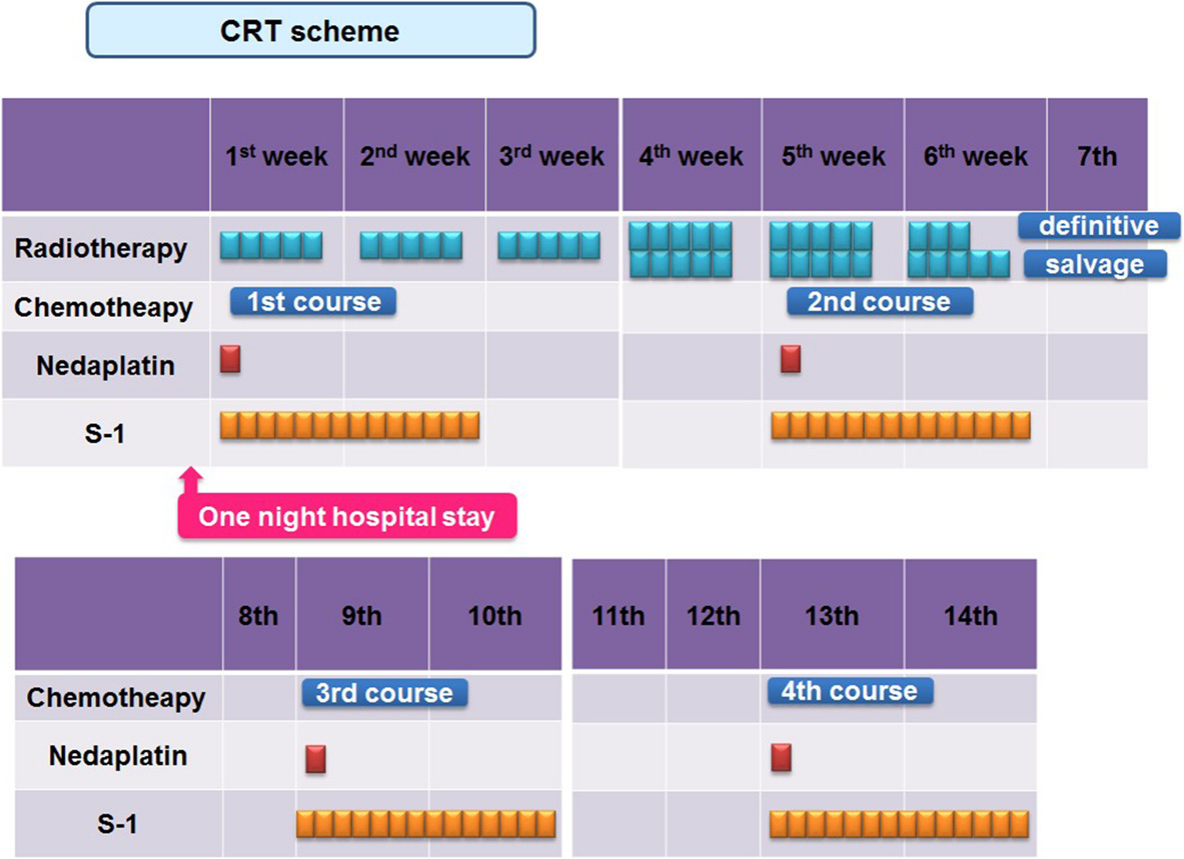
Nedaplatin and S-1 combined with radiotherapy regimen treatment schema

In consideration of concurrent radiation therapy, S-1 was used at a dose of 80 mg/m^2^/day on days 1 through day 14, followed by a 2-week rest, and NDP was delivered intravenously at 80 mg/m^2^ on day 1 every 4 weeks. After the CRT, the chemotherapy was repeated up to a total of four cycles in order to maintain the response or to extinguish the residual tumor (Fig. 1).

In our institution, NDP plus S1 have been administered for all patients regardless of renal or cardiac function since August 2011. Standard regimen of CDDP plus 5-FU has never been performed over this time period.

### Radiotherapy planning

All patients receiving definitive CRT were treated with 50.4 Gy delivered over 5.6 weeks at 1.8 Gy per fraction or 50 Gy in 25 fractions over 5 weeks. No elective irradiation on the mediastinal lymph nodes was performed. Enhanced computed tomography (CT) and/or PET and endoscopic extension were used to define gross tumor volume (GTV) for each patient. All LNs with a diameter at least 1 cm in short axis in CT or positive by ^18^FDG-PET (excluding physiological accumulation) were included in the GTV. The positive was defined as more than 2.5 of standardized uptake value-max. Clinical target volume (CTV)-GTV margin was 2 cm in the cranio-caudal direction and none in the other four directions for the primary tumor. CTV was equal to GTV for metastatic lymph node. We calculated the internal target volume (ITV) which integrated the respiratory motions of CTV in each patient using 4D-CT [12]. Planning target volume (PTV)-ITV margin was 0.5 cm in all six directions.

All patients on salvage CRT received involved field irradiation that covered only GTV plus margin and were treated with 60 Gy delivered over 6 weeks at 2 Gy per fraction. The CTV-GTV margin was 0.5 cm in all directions. The way how ITV and PTV were created was same as the above for definitive CRT cases.

The details of radiotherapy planning and target volume definition of both definitive CRT and salvage CRT were discussed in our previous reports [13–15]. 3D conformal radiation therapy was used to treat these patients.

### Patients

The stage of esophageal cancer was classified according to the UICC version 7 [16]. For staging, upper gastrointestinal endoscopy, X-ray fluoroscopic examination with barium or gastrografin contrast, enhanced CT, and fluoro-deoxy-glucose (FDG)-PET were performed before definitive CRT or radical surgery.

We defined the indications for CRT using this regimen as (a) lower and upper age limits of 20 years and 85 years, respectively; (b) histopathologically proven squamous cell carcinoma or adenocarcinoma of esophagus; (c) clinical stage I without indication for endoscopic sub-mucosal dissection or endoscopic mucosal resection, clinical stages II–III, clinical stage IV consisting of metastases in the supraclavicular/abdominal para-aortic lymph nodes in the definitive CRT group and locoregional oligo recurrence of esophageal cancer after radical surgery in the salvage CRT group; (d) Karnofsky-performance status >70 %; (e) white blood cell counts of 4,000–12,000/mm^3^, neutrophils >2,000/mm^3^, platelets >100,000/mm^3^, hemoglobin >9.0 g/dL, total-bilirubin <1.5 mg/dL, glutamate oxaloacetate transaminase/glutamate pyruvate transaminase <76/72 U/L, serum creatinine value <1.2 mg/dL (creatinine clearance values were not used as inclusion/exclusion criteria), and partial pressure of arterial oxygen >70 mmHg.

The exclusion criteria were: (a) presence of serious complications including fresh gastrointestinal bleeding, active infection, heart failure, renal insufficiency, liver failure, or uncontrolled diabetes; (b) presence of active overlapping cancer; (c) metastasis to other organs from esophageal cancer; (d) a history of RT for the same lesion; (e) contra-indication to receive NDP/S-1; (f) hypersensitivity to NDP/S-1.

### Follow-up method

Blood counts and laboratory tests were performed once a week during CRT. Recurrence was monitored by measuring the levels of the serum tumor markers carcino-embryonic antigen (CEA), squamous cell carcinomarelated antigen (SCC), cytokeratin 19 fragment (CYFRA), p53 antibody every month after completion of treatment and upper gastrointestinal endoscopy (+/−biopsy) plus enhanced CT scan from the upper neck lymph node to the bottom of the pelvis scheduled every three months. When a recurrence was questionable by any of the above examinations, FDG-PET was also performed. We are using Common Terminology Criteria for Adverse Events version 4.0 to measure toxicity.

### Statistical analysis

The Kaplan–Meier method was used for estimation of overall survival, loco-regional control, and disease-free survival. The times for survival were calculated from the start of RT. 95 % CI was calculated by +/−1.96 × standard error.

## Results

### Demographics

The characteristics of the 89 patients (72 males and 17 females) are listed in Table 1. The median age was 65 years, ranging from 44 years to 86 years. The tumor histology was squamous cell carcinoma in 86 patients and adenocarcinoma in the other three patients. The sub-sites of the primary tumors included cervical/upper/middle/lower thoracic portions, with the following distribution: 6/17/52/26 %, respectively. Clinical stage I comprised 21 %, II–III 67 %, and IV 12 % in the definitive CRT group. Among all of the patients, 20 % were 75 years old or older. K-PS before treatment was not over 80 % in 16 cases. Supraclavicular LN metastasis was seen in 4 cases and abdominal para-aortic LN metastasis in 4 cases, although these metastases involved distant and not regional LNs.

**Table 1:** Patient and tumor characteristics

Characteristics	No.	%
Age		
Median	65 years	
Range	44–86 years	
Gender		
Male	72	80.9
Female	17	19.1
Karnofsky performance status		
≧ 90 %	72	80.9
< 90 %	17	19.1
Location of tumor		
Cervix	5	5.6
Upper thorax	15	16.9
Middle thorax	46	51.7
Lower thorax	23	25.8
Clinical T stage		
cT1	22	24.7
cT2	7	7.9
cT3	34	38.2
cT4	26	29.2
Clinical N stage		
cN0	26	29.2
cN1	29	32.6
cN2	23	25.8
cN3	11	12.4
Clinical M stage		
cM0	81	91.0
cM1	8	9.0
Histology type		
Adenocarcinoma	3	3.4
Squamous cell carcinoma	86	96.6

### Compliance with CRT

Twenty-one (24 %) patients completed four cycles, and 94 % received two or more cycles (Table 2). The prescribed full dose and complete cycles of chemotherapy were administered to 64 patients (72 %) according to the pre-treatment planning. The relative dose intensity (RDI) of chemotherapy was 0–24 % in one case, 25–49 % in 2 cases, 50–74 % in 4 cases, 75–89 % in 10 cases, and 90–100 % in 72 cases. Sixty-two subjects (70 %) received treatment as outpatients. S-1 of the second cycle was changed into 5-FU injectable solution in one patient, because the fatigue and languor due to S-1 were severe. This decision was based on our previous experience of using NDP plus 5-FU in definitive CRT for esophageal cancer. As a result, these symptoms improved after changing to 5-FU.

**Table 2:** Chemoradiation (CRT) details and outcomes

	No.	%
Intent of CRT		
Definitive	67	75.3
Salvage	22	24.7
Radiation total dose		
19.8Gy	1	1.1
50Gy	9	10.1
50.4Gy	57	64.1
60Gy	22	24.7
NDP/TS-1 total cycles		
One	5	5.6
Two	60	67.4
Three	3	3.4
Four	21	23.6
Hospitalization during CRT		
Median	2 days	
Range	0–50 days	
Post-CRT morbidity		
Grade 5	1	1.1
Grade 4	2	2.2
Grade 3	4	4.5
State at censoring		
Alive without disease	49	55.1
Alive with disease	13	14.6
Dead of disease	13	14.6

Only one patient (1.1 %) could not complete the planned RT and discontinued treatment at the time of 19.8Gy in 11 fractions (Table 2). This patient had undergone surgery for benign colon disease a few months earlier and the colon wall was perforated, so CRT was suspended at this time. Seventeen patients (19.1 %) received RT up to 60Gy in 30 fractions (Table 2).

### CRT toxicity

The worst toxicities that occurred during the treatment period were grade 3-4 toxicities of neutropenia, leukopenia, anemia, and thrombocytopenia, which occurred in 58, 64, 27, and 27 % of patients, respectively. Grade 4 neutropenia, leukopenia, thrombocytopenia, and anemia occurred in 17, 12, 7, and 10 % of patients, respectively. Five patients developed febrile neutropenia. Grade 3 non-hematological toxicity included infection in 12 %, mucositis/esophagitis in 3 %, kidney in 3 %, fatigue in 3 %, diarrhea in 1 %, and liver function in 1 % of patients (Table 3). Late grade 3 radiation pneumonitis that needed administration of steroids and grade 3 esophageal stenosis were seen in two patients and two patients, respectively (Table 2). Late grade 4 cerebral infarction occurred in one patient. Therapy-related myelodysplastic syndrome developed in one patient. There was only one treatment-related death, which was heart failure at 37 days after start of CRT. This patient had pre-existing angina pectoris.

**Table 3:** Toxicity to chemoradiation by CTCAE v.4.0

	All grades		Grade 3		Grade 4	
	No.	%	No.	%	No.	%
Neutropenia	82	92.1	37	41.6	15	16.9
Febrile neutropenia	–		5	5.6	0	
Leukopenia	83	93.3	46	51.7	11	12.4
Thrombocytopenia	78	87.6	18	20.2	6	6.7
Anemia	88	98.9	15	16.9	9	10.1
Nausea	25	28.1	0		0	
Emesis	8	9.0	0		0	
Diarrhea	18	20.2	1	1.1	0	
Mucositis	64	71.9	3	3.4	0	
Alopecia	0		0		0	
Skin	30	33.7	0		0	
Neuropathy	9	10.1	0		0	
Fatigue	68	76.4	3	3.4	0	
Liver	51	57.3	1	1.1	0	
Cardiac toxicity	1	1.1	0		0	
Pulmonary embolism	0		0		0	
Kidney	25	28.1	3	3.4	0	
Infection	44	49.4	11	12.4	0	

CTCAE = common toxicity criteria

### Efficacy

**Fig. 2:**
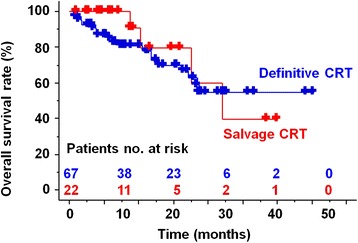
Overall survival curves by the Kaplan–Meier method in definitive and salvage CRT

The median follow-up time of 65 living patients was 11.2 months (range: 1.3–47.2 months). Two patients received salvage sub-total esophagectomy for loco-regional recurrence after definitive CRT. At the end of follow-up, 31 patients (35 %) experienced disease recurrence or local residual disease. The estimated 2- and 3-year overall survivals according to the Kaplan–Meier method were 62.9 % (95 % CI; 47.8–78.0 %) and 54.4 % (95 % CI; 37.3–71.5 %), respectively, in definitive CRT and 59.7 % (95 % CI; 20.9–98.5 %) and 39.8 % (95 % CI; 0–80.8 %), respectively, in salvage CRT (Fig. 2). The estimated 3-year overall survivals in definitive CRT were 73.8 % (95 % CI; 46.8–100 %) in stage I, 47.8 % (95 % CI; 27.4–68.2 %) in stage II-III, and 75.0 % (95 % CI; 32.5–100 %) in stage IV. The estimated 2- and 3-year loco-regional control rates for all 89 patients were 70.6 % (95 % CI; 57.5–83.7 %) and 57.9 % (95 % CI; 38.5–77.3 %), respectively, and the estimated 2- and 3-year disease-free survival rates for all 89 patients were 51.2 % (95 % CI; 37.5–64.9 %) and 39.9 % (95 % CI; 22.1–57.7 %), respectively.

## Discussion

A combination of radiation therapy (RT) with chemotherapy for esophageal cancer in randomized trials has been used to try to improve local control and survival, and cisplatin (CDDP) and 5-FU seem to be the key drugs in these treatment protocols [17–19]. NDP is a platinum derivative that was developed with the aim of reducing renal toxicity while maintaining the effectiveness of CDDP. In an in vivo study, a combination of NDP and 5-FU has been shown to be as effective as a combination of CDDP and 5-FU. In a clinical study, combination chemotherapy using NDP and 5-FU has been reported to be a safe and effective method for treating advanced esophageal cancer. Based on these facts, when the patient’s renal or cardiac function was bad, we had given NDP in place of CDDP since 2000 (i.e. NDP plus 5-FU regimen which is different from NDP plus S1 of this study), because NDP has less renal toxicity than CDDP and because the vigorous hydration needed to safely protect against cisplatin-related nephrotoxicity might affect cardiac function. Radiation combined with NDP and 5-FU was a safe and effective method for treating esophageal cancer. In our previous study [4], a combination of NDP plus 5-FU was comparable to that of CDDP plus 5-FU in survival, loco-regional and distant control, clinical response, and both acute and late toxicity, despite the NDP group had significantly more stage IVB patients (more than 50 %) than did the CDDP group. This chemotherapy regimen was NDP/5-FU and not NDP/S1 as in the present cohort.

The current guidelines such as those of the European Society for Medical Oncology (ESMO) [20] and National Comprehensive Cancer Network (NCCN) (Version 3. 2015) regard carboplatin/paclitaxel as an alternative non-fluoropyrimidine regimen. In many countries, this treatment is considered a standard alternative treatment for concurrent CRT as described in the CROSS (Chemoradiotherapy for Oesophageal Cancer Followed by Surgery Study) trials [21]. Additionally, oral capecitabine can be used as an alternative to 5-FU according to NCCN guidelines. However, in Japan, carboplatin, paclitaxel, and oral capecitabine are currently not included by insurance adaptation in CRT for esophageal cancer with definitive intent. On the other hand, both S1 and NDP are within insurance coverage for esophageal cancer. The costs of NDP plus S1 in Japan are at least comparable to other drug regimens such as capecitabine plus CDDP or carboplatin plus paclitaxel. The carboplatin plus taxane regimen may be also useful as an alternative in terms of efficacy and toxicity in the definitive CRT, although this regimen has been tested mainly in the neoadjuvant setting [21].

Tanaka et al. [22] performed a phase I dose-escalation study on docetaxel, NDP, and S-1 chemotherapy without RT for advanced esophageal carcinoma with T3-4 tumors and/or M1 staging and esophageal carcinoma with cervical lymph node metastasis. There are no other clinical studies published on NDP treatment in combination with S-1.

Minsky et al. [23] reported the activity and toxicity of definitive CRT with 5-FU and CDDP in 218 patients with T1-4/N0-1 esophageal cancer. Their chemotherapy consisted of 5-FU at a dose of 1,000 mg/m^2^/day on days 1–4, 29–32, 57–60, and 85–88 plus CDDP at a dose of 75 mg/m^2^ on days 1, 29, 57, and 85. In 109 patients with standard dose of 50.4 Gy in 28 fractions, acute grade 3–5 toxicity was seen in 77/109 patients (71 %) and late grade 3–4 toxicity was seen in 37/99 patients (37 %) [23]. In their study, the 2-year OS was 40 %, median survival time was 18.1 months, and loco-regional failure plus residual was 52 % [23]. Ishikura et al. [24] assessed the long-term toxicity after definitive CRT for 139 patients with esophageal squamous cell carcinoma. The CRT consisted of two cycles of CDDP 40 mg/m^2^ on days 1 and 8, and continuous infusion of 5-FU 400 mg/m^2^/d on days 1 to 5 and 8 to 12, repeated every 5 weeks with concurrent radiotherapy of 60 Gy in 30 fractions. Of 78 patients with complete remission, two patients died as a result of acute myocardial infarction and grade 3–4 late toxicities occurred with pericarditis in 10.3 %, heart failure in 2.6 %, pleural effusion in 10.3 %, and radiation pneumonitis in 3.8 % of the patients [24]. These rates were almost equal to ours (Table 3), although these comparisons might not be valid because their inclusion criteria were different from ours.

The radiation doses to the fresh cases were different from those administered to recurrent tumors in our institution because we wanted to determine promptly whether there are residual tumors and salvage surgery for them should be adapted after definitive CRT, and we stopped it in the 50–50.4 Gy dose range. The limitations of this report were that this is a retrospective review and that the treatments were not relatively homogeneous in terms of including older patients and patients in both definitive and salvage settings.

Most importantly, NDP plus S-1 can be administered on an outpatient basis, whereas CDDP plus 5-FU, which was used as the standard drug combination in CRT, requires hospitalization of at least 4-5 days per cycle during 5-FU administration. In this retrospective research, 30 % of the patients were treated as inpatients, at least transiently. Twelve of these patients (13 %) were hospitalized up to 2 weeks and 10 patients (11 %) were hospitalized throughout RT. The majority of the admitted patients were admitted because their homes were too far away from the hospital, so it was difficult to commute, or because of severe symptoms of esophageal stenosis by the primary tumor.

## Conclusion

NDP and S-1 in combination with radiotherapy is a feasible treatment, and toxicity is tolerable. This treatment method has the potential to shorten hospitalization without impairing the efficacy of CRT.
